# Correction to: Reconstruction of Microbial Haplotypes by Integration of Statistical and Physical Linkage in Scaffolding

**DOI:** 10.1093/molbev/msaf268

**Published:** 2025-11-04

Chen Cao, Jingni He, Lauren Mak, Deshan Perera, Devin Kwok, Jia Wang, Minghao Li, Tobias Mourier, Stefan Gavriliuc, Matthew Greenberg, A Sorana Morrissy, Laura K Sycuro, Guang Yang, Daniel C Jeffares, Quan Long, Reconstruction of Microbial Haplotypes by Integration of Statistical and Physical Linkage in Scaffolding, *Molecular Biology and Evolution*, Volume 38, Issue 6, June 2021, Pages 2660–2672, https://doi.org/10.1093/molbev/msab037

Figure 6A is an erroneous duplicate of Figure 6B. Figure 6A should describe EHHS decay around each SNP position of the Gag gene of HIV haplotypes, not the Pol gene. The correct version of Figure 6 is shown below.

**Figure:**
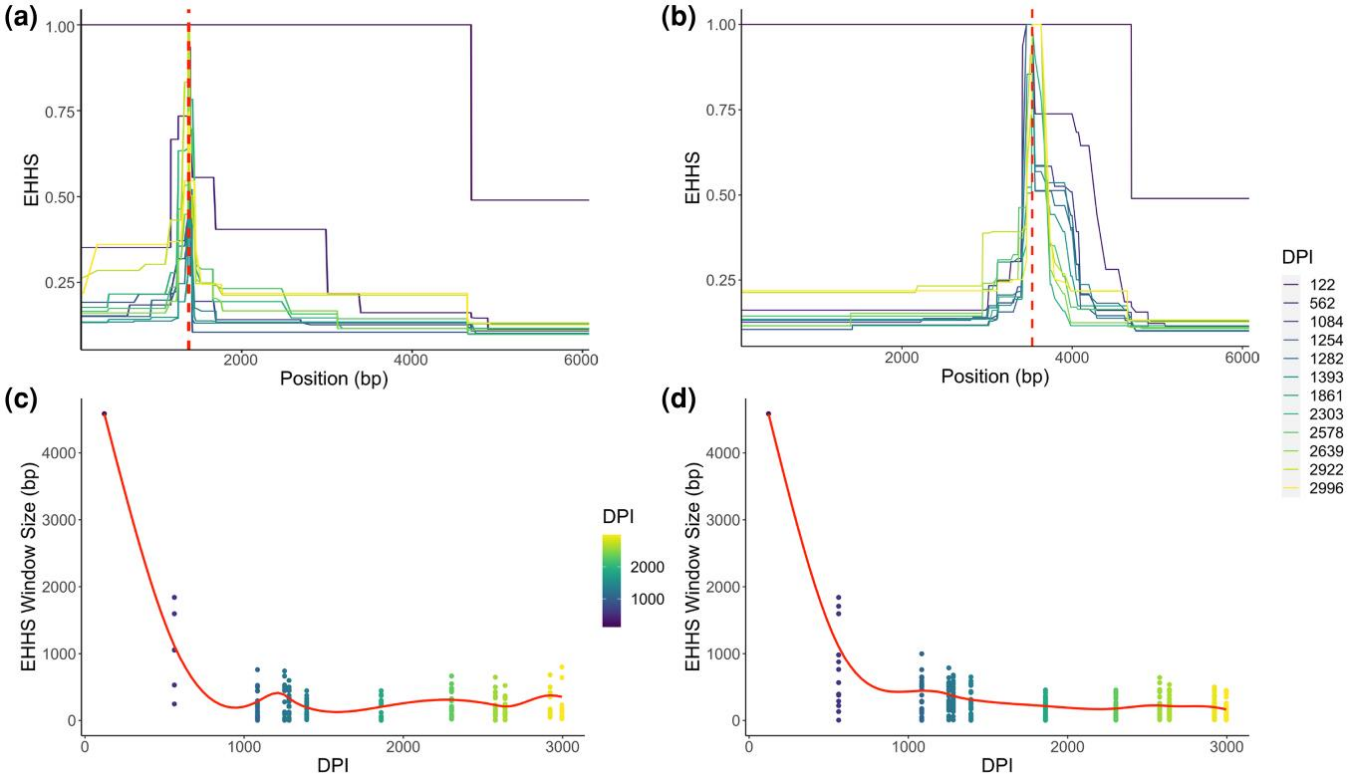


These details have been corrected only in this correction notice to preserve the published version of record.

